# Key principles of miRNA involvement in human diseases

**DOI:** 10.15190/d.2014.26

**Published:** 2014-12-31

**Authors:** Dana Elena Giza, Catalin Vasilescu, George A. Calin

**Affiliations:** Department of Experimental Therapeutics, University of Texas MD Anderson Cancer Center, Houston, TX, USA; Department of Hematology, Fundeni Clinical Hospital, Bucharest, Romania; Department of Surgery, Fundeni Clinical Hospital, Bucharest, Romania; UMF Carol Davila, Bucharest, Romania

**Keywords:** miRNA, microRNA, biomarkers, gene targeted therapy

## Abstract

Although rapid progress in our understanding of the functions of miRNA has been made by experimentation and computational approach, a considerable effort still has to be done in determining the general principles that govern the miRNA’s mode of action in human diseases. We will further discuss how these principles are being progressively approached by molecular studies, as well as the importance of miRNA in regulating different target genes and functions in specific biological contexts. There is a great demand to understand the principles of context-specific miRNA target recognition in order to design future experiments and models of normal developmental and disease states.

## 1. Introduction

Micro ribonucleic acids (miRNAs) are a group of small, noncoding RNA molecules with 20–22 nucleotides (nt) in length that are able to control the activity of up to 30% of all protein‐coding genes in mammals^1^. As our understanding of the biogenesis and mechanisms of action of miRNAs has grown over the past decade, new information about their importance in fine-tuning the regulation of gene expression turned out to play an important role in various pathological processes, including cancer, cardiovascular disease, diabetes, mental disorders and viral infection^2^.

Using high-throughput genomics and bioinformatics, miRNA research is now positioned to make the transition from laboratories into clinical studies, offering the promise that miRNAs can serve as valuable therapeutic targets for a large number of diseases. Therefore, the use of miRNAs as therapeutics is without doubt the next frontier in treatment options for several human diseases. Herein, we overview the key principles of the miRNA’s involvement in human diseases, as well as the potential for miRNA to become the next generation tools used in diagnostics and therapeutics.

## 2. MicroRNAs regulate gene expression

microRNAs are a class of small non-coding RNAs that can regulate gene expression by mechanisms that are still not fully understood or explored and which include messengerRNA degradation, translation inhibition, promoter binding, protein binding, or direct interaction with other non-coding RNAs.

microRNAs are encoded in the genome as pre-miRNAs, long primary transcripts that contain a cap structure at the 5’ end and are polyadenylated at the 3’ end. The miRNA biogenesis starts with the processing of the pre-miRNAs by the cellular endonuclease III Drosha together with DGCR8/Pasha into precursor-miRNA (pre-miRNA), which are then exported from the nucleus to the cytoplasm by an Exportin 5-dependent mechanism. The pre-miRNA is then cleft by the RNase III enzyme Dicer in cytoplasm, producing a short imperfect double stranded miRNA duplex. The miRNA duplex is further unwound in a mature miRNA that will be incorporated in RISC, which is a complex constituted by the components of the Argonaute family protein^3^.

The function of miRNAs is to inhibit the protein synthesis of protein coding genes by inhibiting the translation of or by degrading the mRNA^3^. Although the relative contribution of each of the miRNAs mechanism of gene regulation is still unknown, it is believed that mRNA destabilization is the predominant mechanism of action to decrease the target levels. Moreover, it was shown that the miRNAs mechanisms of action suffer plasticity, in the sense that miRNAs can also activate translation of targeted mRNAs, switching between translation repression and activation in coordination with the cell cycle^4^.

Most of the studies investigating miRNAs-target interaction focus on the 3’ UTR of mRNAs^5^. Lytle et al brought a new perspective in the study of this interaction suggesting that miRNAs could actually associate with any position of target mRNAs, efficiently repressing them by binding to miRNA-binding site in 5’ UTR^6^. Other mechanisms of action for miRNAs include targeting gene promoters^7^and also decoy activity that interferes with the function of regulatory proteins^8^. Furthermore, miRNAs can also regulate gene expression at the transcriptional level by directly binding to DNA regulatory elements^9^.

## 3. Each miRNA has hundreds or thousands of targets, and probably the full coding genome is under the control of miRNAs

Most experimental approaches focus on identifying miRNA targets and investigating how the expression of each miRNA can control the expression of tens or hundreds of mRNA due to imperfect complementarity, while several miRNAs can control a single mRNA. As these small miRNA molecules regulate a significant part of the mammalian transcriptome, recent published studies suggest that they are organized in gene regulatory networks that are larger than previously believed^10^. An increasing effort has been made in investigating these complex gene regulatory networks and their role in normal and pathological conditions. As the literature suggests, miRNA molecules play important roles in physiological processes like cell growth, differentiation, proliferation, apoptosis, signal transduction pathways^11-13^. As key players regulating genetic programs and stabilizing developmental pathways, miRNAs have an essential role in reinforcing molecular pathways prone to variations in gene expression. The miRNA’s genes were found to be expressed in many types of physiological processes and pathways, such as B-cell lineage fate (*miR-181*), B-cell survival (*miR-15a* and *miR-16-1*), cell proliferation control (*miR-125b* and *let-7*), brain patterning (*miR-430*), pancreatic cell insulin secretion (*miR-375*) and adipocyte development (*miR-145*)^14^. Moreover, changes in miRNA’s expressions occur in response to environmental stimuli and play an important role in regulating the immune response, inflammation primarily, by regulating the pathways associated with the nuclear factor kappa beta (NF-kB), the central mediator of inflammatory response^15^. These findings not only provided insights about miRNA mediated inflammatory responses, but also inspired researchers to look at miRNAs as potential drug targets for fine-tuning the immune system. Aberrant miRNA expression should proportionally affect these critical processes leading to various pathological outcomes. A more comprehensive profiling of the miRNA expression will be able to identify the miRNA patterns that hold great prognostic values for a specific disease.

## 4. MicroRNAs are involved in the pathophysiology of human diseases

**Figure 1:**
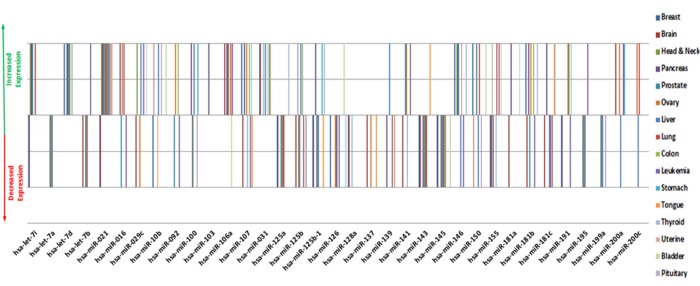
miRNA abnormally expressed in human cancers - examples from several different publications

An increasing number of studies have shown that miRNAs are involved in the development and progression of a variety of diseases^16^ (**Table 1**). Mutations, dysregulations or dysfunction of miRNA biogenesis and their targets leads to the blockage of physiological and biochemical pathways involved in the development and the evolution of diseases in humans. Using computational prediction several miRNA-disease associations were identified. Cancer disease genes are more prevalent in miRNA-targeted human disease genes when compared to the other disease gene classes, although the real reason why cancer genes are more targeted by miRNAs is still unknown^17,18^. Differences in the miRNA’s expression between tumors and normal tissues have been identified in lymphoma, breast cancer, lung cancer, papillary thyroid carcinoma, glioblastoma, hepatocellular carcinoma, pancreatic tumors, pituitary adenomas, cervical cancer, brain tumors, prostate cancer, kidney and bladder cancers, and colorectal cancers (see **Figure 1**). It was previously reported that longer gene structure, higher enrichment of AREs, higher duplicability, slower evolutionary rates, and lower mRNA decay rates of cancer genes make cancer genes a good substrate for miRNA targeting^2^. Furthermore, microRNA’s altered expression was also reported in other human, non-cancer diseases including schizophrenia, neurodegenerative diseases like Parkinson’s disease and Alzheimer disease, immune related disease, and cardiac disorders^16^. There is also preliminary evidence that several viruses are capable to regulate the host cellular miRNAs in order to gain control of their survival and they do so also by encoding their own miRNAs, like in the case of polyoma virus, adenovirus, and herpes viruses^19^. These findings may help explain miRNA’s disease-specificity and provide novel targets for therapy.

## 5. microRNoma

microRNoma is defined as the full complement of microRNAs present in a genome. microRNoma alteration in cancer cells expression is characterized by abnormal levels of expression for mature and/or precursor miRNA sequences in comparison with the corresponding normal tissues.

After publishing the first report of the role of miRNAs in cancer, Calin et al. found that a significant percentage of miRNAs is located at fragile sites and altered regions in cancer, including regions of amplification or loss of heterozygosity and breakpoints^20^. A total of 50% of the human miRNAs are localized in fragile chromosomal regions associated with amplifications, deletions, or translocations that occur during tumour development^21^. Various mechanisms were found to be involved, such as the location of miRNAs at cancer associated genomic regions (CAGR), the epigenetic regulation of miRNA expression, abnormalities in miRNA processing genes, the complex regulation of miRs by oncogenes, and tumor suppressor genes^22^. Examples of epigenetic mechanisms that regulate miRNA expression in cancer include the miR-34a,-b and –c family that are induced directly by TP53, by binding to the miR-34s’ promoters^23^, *c-Myc *negative regulation of tumor suppressors miRNAs like let-7 family (let-7a-1, let-7f-1, let-7d, let-7c and let-7g)^24^. This explains in part why there are differences of miRNAs expression in malignant cells versus normal cells, and sometimes within the same tumor cells.

Several steps during tumorigenesis, also defined as cancer hallmarks, are believed to be regulated by miRNAs: (1) abnormal apoptosis, (2) unlimited replicative potential, (3) antigrowth signals insensitivity, (4) induction of angiogenesis, (5) invasion, and (6) metastasis^21^. The mechanisms underlying the above mentioned steps along with the implication of the miRNAs in malignant cell transformation, tissue invasion, and colonization to distant organs still need to be elucidated. The unique pathogenic pattern of miRNA alterations in cancer along with miRNAs-tumor type specificity permits precise tumor identification and classification^25^. miRNA profiling studies demonstrated a direct link between the dysregulation of miR-125b, miR-145, miR-21, and miR-155 expression and an increased risk for breast cancer^26^. Moreover, the up-regulation of miR 155 and down-regulation of the let 7a were correlated with poor survival rates in lung cancer^27^ (**Figure 1**).

## 6. MicroRNAs can act as tumors suppressors (TS-miRNAs) or as onco-miRs

In cancer research, miRNAs were divided in two different categories based on their expression: (1) miRNAs that act as oncogenes (such as *miR-21* and *miR-155*, which cause acute B-cell leukemia in transgenic mice models) and (2) miRNAs that act as TSGs^21^. The same miRNA that is acting like an oncogene in one type of cells can act like a suppressor in another due to different targets and mechanisms of action (miR-222 is hyperexpressed in liver cancers where it targets the PTEN suppressor, while the same miRNA is downregulated in erythroblastic leukemias where it targets the c-KIT oncogene)^28^. For example, miR-15, miR-16, and let-7 are known tumor suppressors while miR-21 and miR-155 serve as oncogenes^21^. miR-15 and miR-16 repress the expression of the anti-apoptotic gene bcl-2 which promotes cell death in cancerous cells and it was found to control the expression of 14% of all genes in the human genome^29^. A role of miRNA acting as oncogene was attributed to miR-155 in early leukemogenesis, proven in a transgenic mouse model with overexpression of miR-155, which underwent a polyclonal preleukemic pre-B cell proliferation followed by full blown B cell malignancy^30^. Other strong evidence for miRNAs acting as oncogenes is represented by the miR-21 upregulation in a variety of hematological malignancies (AML, CLL) and solid tumors (gliobaslatoma, pancreas cancer, prostate, stomach, colon, lung, breast, and liver)^31^ and miR-17-92 cluster, which is transactivated by the *c-Myc* oncogene and accelerates lymphomagenesis in mice^32^.

## 7. miRNAs and cancer predisposition

miRNAs may contribute to cancer predisposition and initiation by germline and somatic mutations in active, precursor, or primary miRNAs molecules.

Cancer predisposition and development was shown to be also the result of germline and somatic mutations in active pre- or pri-miRNAs^21^. The initial reports of these sequences variation included the miR-15a/16 cluster mutations that occur in rare families with high incidence of both CLL and breast cancer^20^. When screening for genetic variants of miRNAs that regulate key breast cancer genes, 7 new variants were identified, 2 among them were found in pre-miRNAs (pre-miR-30c-1 and pre-miR-21) and 5 in pri-miRNAs (pri-miR-17, pri-miR-24-1, pri-miR-125a, pri-miR191, and pri-miR-125b-1^33^. Moreover, polymorphisms in the messenger protein coding RNAs targeted by miRNAs can also influence the cancer risk (such as the let-7 complementary SNP site in the KRAS 3' untranslated region which was found to be significantly correlated with an increased risk for non small cell lung carcinoma among moderate smokers^34^.

## 8. MicroRNAs profiling

microRNAs profiling allows identification of specific signatures associated with diagnosis, progression, prognosis, and response to treatment in human diseases.

Although the number of the reports discussing the miRNAs’ involvement in human disease is increasing, the pattern of miRNA-disease association remains largely unclear. A human miRNA association disease network was built by Lu et al^35^, using data manually collected from publications, which provided evidence that miRNAs show similar or different dysfunctional evidence for the similar or different disease cluster. In addition, the authors found that the miRNAs associated with the same disease are organized in pre-defined groups and that there is a negative correlation between the tissue-specificity of a miRNA and the number of diseases it associated with. As a single miRNA can have many potential targets, the translations of many genes in multiple pathways can be simultaneously regulated by a single type of miRNA. Therefore, a relatively small change in the miRNA expression might lead, by collectively adding, to large changes in physiological but also pathological states ranging from infection to cancer.

Although the discovery of the miRNA-regulatory circuits is still in its infancy, the identification of specific miRNAs-disease signature could provide the rationale for disease classification, early detection, and therapeutic options. The extensive genome wide expression profiling of cells and tissues in different stages of differentiation, metabolic conditions, and disease models are very specific for the types of samples studied. Furthermore, the miRNA-based classifier was found to be more useful in establishing the correct diagnosis in the metastatic cancer of unknown primary site than the coding genes messenger RNA classifier. Poorly differentiated tumors have lower global miRNA expression levels when comparing with well-differentiated tumors from control groups. Therefore, the reduced expression levels of miRNAs in poorly differentiated tumors is probably the main reason why miRNA profiling is effective in the diagnosis of cancer of unknown primary site^21^**. **In this sense, a specific miRNA expression signature of 13 miRNAs was identified in CLL, associated with the disease progression from the time of diagnostic to the time of therapy^20^. Similarly, a miRNA expression pattern was correlated with the incidence, prognosis, and survival rate of patients with acute myeloid leukemia^36^.

## 9. miRNAs as biomarkers

Measurement of miRNAs in body fluids including plasma and serum may represent a gold mine for noninvasive biomarkers in cancer.

The ultimate goal of molecular and computational approaches of the last decade in miRNAs research was to reveal the functions of miRNAs in human disease and to establish their role as biomarkers able to improve diagnosis and prognosis of disease. miRNAs can be considered early biomarkers due to their upstream positions in the regulation cascades^16^. Moreover, they can be easily identified using genomic tools such as oligonucleotide microarrays and deep sequencing which deliver higher throughput than mass spectrometry used for protein and metabolite biomarker identification.

miRNAs represent a novel attractive diagnostic biomarker due to their higher stability when compared to RNAs and remain stable after being subjected to severe conditions that would normally degrade most RNAs, such as boiling, very low or high pH levels, extended storage, and 10 freeze-thaw cycles^37^. Even in low abundant expression, miRNA can be amplified and then detected in a clinical setting by real-time quantitative PCR (qPCR), an approach used in FDA-approved clinical tests already. The adoption of the locked-nucleic acid (LNA) technology in miRNA probe design could improve the sensitivity and specificity of miRNA qPCR assays even further^38^.

MiR-146a, miR-150, miR-223, miR-574-5p and a panel of 6 plasma miRNAs were found already to be potential biomarkers for sepsis and systemic inflammatory response syndrome (SIRS)^39^. Recent published data clearly suggested that miRNAs profiling is useful in identifying predictive miRNA signatures associated with several cancer types like pancreatic cancer, colorectal cancer, cell renal cell carcinoma, and osteosarcoma^40^. The changes in miRNAs expression can be also detected from circulating tumor cells in blood samples/plasma and urine or by tumor slide-based staining. Furthermore, correlations between circulating miRNA expression levels and response to a given anticancer treatment have already been made. Serum *miR-21* levels were higher in hormone-refractory prostate cancer patients whose disease was resistant to docetaxel-based chemotherapy when compared to those with chemosensitive disease^41^.

## 10. miRNAs as targeted therapies

The ability of miRNAs to target genes from the same pathway and/or in interacting pathways is one of the main reasons to use a small number of miRNAs to achieve an orchestrated broad silencing of pro-tumoral pathways. Therefore, miRNA therapeutics might be superior to a mixture of small interfering RNAs (siRNAs) that are specifically designed to reduce the expression of a given number of target genes. The rationale of using miRNAs as anticancer drugs was the results of the observation that miRNA expression is deregulated in cancer compared with normal tissues and that cancer phenotype can be changed by targeting miRNA expression. miRNA therapeutics is focused on downregulating or blocking the function of oncogenic miRNAs or to upregulate the expression of miRNAs that have a tumour-suppressive function^17^. There are two main strategies to target miRNA expression in cancer, which include (1) direct strategies, involving the use of oligonucleotides or virus-based constructs to either block the expression of an oncogenic miRNA or to substitute for the loss of expression of a tumor suppressor miRNA and (2) indirect strategies involving the use of drugs to modulate miRNA expression by targeting their transcription and their processing^42^. Moreover, molecular approaches are trying to reverse epigenetic silencing or to enhance the biogenesis of miRNAs and levels of silenced or deleted miRNAs can be restored by the direct administration of miRNA formulations - naked, coupled to a carrier, or delivered via a viral vector. Strategies that block miRNA functions, both oligonucleotide-based and small-molecule-based approaches are currently being explored.

The advantages of using miRNAs are: first, miRNAs are “natural” products produced in human cells in contrast to chemotherapies or antisense oligonucleotide and, second, microRNAs target multiple genes from the same pathway. The first miRNA drug development was a LNA-modified oligonucleotide, SPC3649 developed by Santaris Pharma A/S, in order to repress the expression of miR-122, in treating chronic hepatitic C virus (HCV) infection with an impressive repression efficacy on miR-122 in mice^15^. Compared to a combined administration of pegylated interferon-a and ribavirin, the standard treatment for HCV infection, SPC3649 demonstrated better safety profiles in chimpanzees and desired tolerance in healthy volunteers.

## 11. Other types of non-coding RNAs

New categories of not-translated RNAs such as lincRNAs and ultraconserved genes were found to be abnormally expressed in cancer and to be involved in tumorigenesis.

The estimates of non coding RNAs (1,000,000 ncRNA transcripts) is much wider as of miRNAs (10,000 potential microRNAs), therefore their impact on basic and translational research is important^42^. lncRNAs, as a member of the non coding RNA family, are defined as RNA molecules that are longer than 200 nucleotides which are not translated into proteins. They can be further subdivided based on their structural or functional characteristics, into circular RNAs (circRNAs), natural antisense transcripts (NATs), transcribed ultraconserved regions (T-UCRs), long enhancer ncRNAs, long intergenic ncRNAs (lincRNAs) and pseudogenes^42^. Their expression has been linked to many physiological processes such as X-chromosome inactivation, cell differentiation, immune response and apoptosis, and to various steps in tumorigenesis like sustaining proliferative signaling; evading growth suppressors; enabling replicative immortality; activating invasion and metastasis; inducing angiogenesis; resisting cell death; and reprogramming energy metabolism^43^.

lncRNAs, as well as miRNAs, have been divided in cancer research into oncogenic and tumor-suppressor lncRNAs^43^. Their high stability, tissue specificity, and efficient detection in body fluids enable them to serve as novel biomarkers for diagnostic, prognostic, and monitoring purposes. Examples of lncRNAs involvement in cancer are represented by HOX transcript antisense RNA (*HOTAIR*), a 2.2 kb lincRNA residing in the *HOXC* locus, which was found to be highly expressed in breast cancer samples^44^and the adenocarcinoma transcript 1 (*MALAT1*), that was found to predict metastasis and survival in early-stage NSCLC^45^. NATs are transcripts encoded in the genome with sequence complementarity to protein-coding RNA transcripts which regulate approximately one-third of protein-coding genes^46^. The regulatory mechanism of NATs is considered to be the result of epigenetic modulation (such as DNA methylation induced by the interaction of NATs with DNA methyltransferases). Another representative of the lncRNAs is a cancer-associated transcript 2 (*CCAT2*), a T-UCR that is transcribed from the highly conserved 8q24 cancer risk locus and is upregulated in microsatellite-stable colorectal cancers, which was shown to promote oncogenic activity and induce chromosomal instability in colorectal cancers^47^.

Recently, a new concept of gene regulation by competitive endogenous RNAs (ceRNAs) has emerged suggesting that mRNA targets have an active role as key elements in regulating miRNA availability within cells^48^. Unlike artificial miRNA sponges, ceRNAs, which are considered to be natural decoys of miRNA activity, include transcripts such as pseudogenes, long non-coding RNAs (lncRNAs) and mRNAs. ceRNAs compete for a common set of miRNAs and are believed to be natural decoys in the gene regulation.

The low levels of lncRNA expressed only by a selected subpopulation of cells^49^ render a specific expression pattern of lncRNAs in certain types of tissues or cells and provide a unique opportunity for specific regulation by lncRNA-targeting therapeutics. Various lncRNA therapeutics are being investigated and several companies are also actively developing lncRNA-targeting therapeutics for the treatment of human diseases^50^.

**Table 1:** miRNAs expression in human diseases

Disease	MiRNA expression	Reference
Cancer		
B-CLL	miR-15, miR-16	^51^
Breast cancer	miR-125b, miR-145, miR-21, miR-155, miR-210	^22,26,27^
Lung	miR-155, let-7a,	^27^
Gastric cancer	miR-145	^52^
Liver cancer	miR-29b	^53^
Infectious diseases		
HCV	miR-122, miR-155	^15,54^
HIV-1	miR-28, miR-125b, miR-150, miR-223, miR-382	^55^
Influenza virus	miR-21, miR-223	^56^
Immune mediated pathology		
Multiple sclerosis	miR-145, miR-34a, miR-155, miR-326	^57^
Systemic lupus erythematosus	miR-146a	^58^
Type II diabetes	miR-144, miR-146a, miR-150, miR-182, miR-103, miR-107	^59^
Nonalcoholic fatty liver disease	miR-200a, miR-200b, miR-429, miR-122, miR-451, miR-27	^60^
Non-alcoholic steatohepatitis	miR-29c, miR-34a, miR-155, miR-200b	^61^
Neurodegenerative diseases		
Parkinson’s disease	miR-30b, miR-30c, miR-26a, miR-133b, miR-184*, let-7	^62^
Alzheimer’s disease	miR-29b-1, miR-29a, miR-9	^63^

## 12. Conclusions

The study of miRNAs’ functions has revealed a new regulatory layer that might be significant given the amount of missing information about the genetic and epigenetic variability in the etiology and pathology of human diseases. These new genome-scale regulatory networks of miRNAs can be considered valuable disease patterns which allow researchers to modulate miRNA expression in response to usual therapy and to further develop novel therapies. Using the large quantity of integrated bioinformatics data collected to date, one could analyze the specific miRNA-disease association in order to put an early diagnostic and to develop personalized therapeutic strategies based on the miRNA’ specific signature.

## KEY POINTS

**◊** **microRNAs are small transcripts that regulate the expression of the majority of protein coding genes**

**◊** **microRNAs abnormalities were identified in any type of disease analyzed to date**

**◊** **microRNA expression could be used for predictive signatures of survival and response to therapy in many disorders, including cancers**

**◊** **the development of miRNA-based gene therapy is taking shape in clinical practice**
